# Anesthetic Considerations in a Patient with Amiodarone-Induced Thyrotoxicosis

**DOI:** 10.1155/2010/984981

**Published:** 2010-06-10

**Authors:** Paul Calis, Remco Berendsen, Angelique Logeman, Elise Sarton, Leon Aarts

**Affiliations:** Department of Anesthesiology, Leiden University Medical Center, 2333 ZA Leiden, The Netherlands

## Abstract

Amiodarone-induced thryrotoxicosis (AIT) is a rare but serious complication of amiodarone use, especially in patients with severe cardiac disease. We present a patient who developed AIT, following administration of amiodarone for life-threatening ventricular arrhythmias. We discuss the medical management of AIT and anesthetic considerations for management of patients with thyrotoxicosis and severe cardiac disease who require surgery including thyroidectomy.

## 1. Introduction

Amiodarone is an effective antiarrhythmic drug. It has a number of side effects including thyroid dysfunction, due to its high iodine content and direct toxic effect on the thyroid. Amiodarone is frequently used in patients with supraventricular and ventricular arrhythmias [1]. 

AIT is rare but seen in patients with severe cardiac disease since it is predominately used in this population. AIT has multiple effects on a patient's physical condition including on cardiac performance. Current treatment options include medical therapy and thyroidectomy. Anesthetic management of patients with AIT and poor cardiac function is challenging. Here, we report the management of a patient with AIT and severe cardiac disease. Anesthetic management including techniques and potential complications is discussed.

## 2. Case Report

A 46-year-old man with idiopathic dilated cardiomyopathy was admitted for the treatment of sustained ventricular tachycardia using radiofrequency ablation. Echocardiography showed left ventricular dilatation, left ventricular ejection fraction of 30%, normal right-sided chambers, and mild mitral regurgitation. Coronary angiogram demonstrated normal coronary arteries. Two years earlier he had been successfully resuscitated following collapse due to ventricular fibrillation and had an implantable cardioverter-defibrillator (ICD) placed. Due to persistent episodes of ventricular tachycardia and ventricular fibrillation, treatment with amiodarone was initiated. Ventricular arrhythmias persisted and attempts were made at radiofrequency ablation, but were unsuccessful. Cryoablation by way of sternotomy was successfully performed, although the postoperative period was complicated with mediastinitis requiring antibiotic treatment and vacuum-assisted closure of the wound. Several days postoperatively the patient complained of a fine tremor of the hands and tachycardia. Thyroid function tests (TFTs) demonstrated an increased free T4 of >99.9 pmol/L (10–24 pmol/L), a normal T3 of 2.5 nmol/L (1.1–3.1 nmol/L), and a decreased TSH of <0.005 mU/l (0.3–4.8 mU/L). The diagnosis AIT was made. Amiodarone therapy was stopped and the patient started on propylthiouracil. One week later, sodiumperchlorate was added as free T4 and TSH levels were unaltered, but T3 level increased to 6.5 nmol/L. The following week prednisone was added as T3 and free T4 remained high. Metoprolol was increased. Despite receiving triple therapy for AIT, there was no improvement in the TFTs. Due to concerns regarding the duration of hyperthyroidism and its potential effects on cardiac function a decision was made to perform a thyroidectomy. Four weeks after the initial sternotomy, thyroid surgery was performed. Because of the combination of closure of the sternal wound and thyroidectomy and little experience of performing thyroidectomy under locoregional anesthesia, surgery was performed under general anesthesia. The patient was premedicated with lorazepam 2 mg sublingual. Etomidate 0.25 mg/kg and fentanyl 2 mcg/kg were used for induction because of their cardio-stabile profiles. Atracurium 0.5 mg/kg was given to facilitate intubation. Anesthesia was maintained using propofol and remifentanyl, titrated to maintain a bispectral index between 40 and 45%. Morphine 0, 15 mg/kg was given for postoperative pain. No complications were seen during or after surgery. Within several weeks after the procedure, TFTs improved and the patient was started on thyroxine. The patient was discharged to another hospital for rehabilitation.

## 3. Discussion

Amiodarone is a class III antiarrhythmic drug which is widely used for ventricular and supraventricular arrhythmias. Amiodarone has a range of side effects which include interstitial pneumonitis, cardiac disorders, and thyroid dysfunction [1, 2]. Both hypothyroidism and hyperthyroidism are recognized and can occur in up to 25% of patients [3]. The average prevalence for AIT is about 5% with a maximum of up to 27% described [1, 4]. Thyrotoxicosis is diagnosed by clinical signs and thyroid function tests. Symptoms include weight loss, decreased appetite, frequent stools, tremor, emotional liability, and heat intolerance. Physical examination demonstrates tachycardia, fine tremor, thyroid enlargement or nodules, thyroid bruit and myopathy. In AIT tiredness and weight loss predominate due to amiodarone-mediated alpha and beta adrenergic blockade. Thyroid function tests most commonly show a low thyroid-stimulating hormone (TSH) level and high thyroxine and T3 levels. 

Management of AIT is a challenge, involving three therapeutic options [5]. The first involves discontinuing amiodarone use. This may not be an option if there are no further treatment options available for the underlying arrhythmia. One case review states that 5% of the patients treated with amiodarone for an arrhythmia are refractory to other treatments [4]. In addition improvements in thyrotoxicosis take several months after cessation of amiodarone therapy. A rebound rise in T3 and loss of the beta blockade may exacerbate thyrotoxicosis on stopping amiodarone therapy.

The second option is medical treatment, including thionamides, perchlorates, and corticosteroids. Thionamides block hormone synthesis by interfering with iodine organification and the coupling of iodothyrosines. Perchlorates competitively block iodide from entering the thyroid gland. Corticosteroids suppress the effects of autoimmune thyrotoxicosis. Treatment may take as long as 4 months to become effective. Other medical options include radioiodine and iopanoic acid, which inhibits peripheral conversion of T4 to T3. Plasmapheresis can temporarily improve the clinical situation in cases of severe thyrotoxicosis refractory to medical treatment. In addition to antithyroid medication, symptomatic treatment of tachycardia with beta-blockers may be started. Propanolol has the advantage over other beta-blockers of inhibiting peripheral conversion of T4 to T3. Beta-blockers given in combination with cordarone can cause profound bradycardia. Medical treatment can itself cause side effects (see Table 1). 

The third option is surgical treatment. Total or near total thyroidectomy is a definitive treatment in thyrotoxicosis. Thyroidectomy is ideally performed when the patient is euthyroid. The clinical manifestations of thyrotoxicosis can occasionally necessitate surgery prior to an euthyroid state. Thyroid hormones have important influences on cardiac function. Increasing levels of T3/T4 cause increased systemic vascular resistance and isovolumic relaxation time to decrease and heart rate, ejection fraction, cardiac output, and blood volume to increase [6]. Hyperthyroidism predisposes a patient to developing supraventricular arrhythmias, for example, atrial fibrillation. Dyspnea on exertion and fatigue develops in approximately 50% of patients. With hyperthyroidism patients are generally hypovolemic. In patients with pre-existing cardiac disease hyperthyroidism may cause rhythm disturbances, cardiac failure, angina pectoris, and cardiomyopathy [7]. Thyrotoxicosis can progress to a thyroid storm, which is associated with mortality rates of up to 30%. Thyroid storm may be induced by infection, surgery, iodine exposition, trauma, pregnancy, and metabolic disorders. The exact pathogenesis of thyroid storm is not known. Two potential mechanisms are described. A sudden increase of free T4 may occur due to a change in T4 protein binding or altered synthesis of thyroxin binding globulin as occurs in severe disease like sepsis and ketoacidosis. Alternatively changes in sympathetic nervous system activation may induce a thyroid storm. An increase in beta-adrenergic receptor density and modulation of adrenergic receptor expression by thyroid hormone can lead to symptoms of a catecholamine excess [8].

Thyroidectomy in patients with thyrotoxicosis can be performed under general or local anesthesia. General anesthesia provides optimal conditions for the surgeon. It is associated with some rare but serious complications including myocardial infarction, stroke, and even death. General anesthesia has many side effects including nausea, vomiting, disorientation, sore throat, shivering, and dizziness [9]. Patients with thyrotoxicosis should continue all antithyroid medication in the perioperative period. Although beta-blockers are negatively inotropic and should be used with care, its generally advised to control cardiovascular responses evoked by sympathetic nervous system stimulation with beta-blockers [10]. For elective surgery long-acting beta-blockers can be titrated preoperatively until a heart rate less than 100 beats per minute is achieved. For emergency surgery intravenous esmolol can be used to achieve the same goal. Adequate premedication is advised [11, 12]. Oral benzodiazepines are often used for anxiolysis. Induction of anesthesia can be achieved with several intravenous induction agents. Thiopental possesses antithyroid activity but is cardiodepressant. Ketamine should be avoided in patients with thyrotoxicosis because it stimulates the sympathetic nervous system. Neuromuscular blocking agents should be used cautiously as thyrotoxicosis is associated with an increased incidence of myopathies. Pancuronium should be avoided as it is arrhythmogenic [11]. Indirectly acting adrenergic agonists can cause unexpected rises in blood pressure and arrhythmias. Phenylephrine is therefore a more appropriate choice [12]. Monitoring during maintenance of anesthesia is directed at early recognition of thyroid storm. Body temperature can rise and hyperpyrexia should be managed with a cooling mattress and cold intravenous fluids. ECG monitoring may show tachycardia and cardiac dysrhythmias which could be treated with intravenous beta-blockers or lidocaine [11]. In patients with severe cardiac disease, general anesthesia can cause a compromise in cardiac function with its associated intubation, hypotension, and negative inotropy. Several studies show general anesthesia to be safe in patients with AIT and severe cardiac disease. Haemodynamic changes were rare and thyroid storm was never encountered [4, 13]. There was no preferential anesthetic technique showed. 

Use of a local anesthesic technique has the advantage of avoiding the cardiac stress associated with intubation and extubation. The side effects of general anesthesia are prevented. Other benefits include a shorter postoperative recovery period and reduction of costs [9]. Important considerations for using a local anesthesic technique are a cooperative patient, a surgeon accustomed with performing a thyroidectomy under these circumstances, and no allergy to local anesthetics. Contraindications include a known retroesophageal or retrotracheal goitre, concomitant cervical lymphadenectomy, and known or suspected locally invasive cancer [14, 15]. Local anesthesia can be accomplished using a regional block of the cervical plexus combined with an anterior field block or an anterior field block alone. Both are generally combined with conscious sedation and analgesia using short-acting hypnotics such as propofol and midazolam and short acting analgesics such as fentanyl and alfentanil. Spanknebel et al. describe a method of bilateral superficial cervical plexus blockade combined with an anterior field block for thyroidectomy. Good anesthetic conditions were obtained with high patient satisfaction and only a small percentage of patients requiring general anesthesia [15]. Snyder et al. compared local anesthesia and general anesthesia for thyroidectomy and found similar operative results, clinical results, and patient satisfaction [16]. A retrospective study demonstrated that local anesthesia combined with sedation in patients with AIT and severe cardiac disease was safe [17]. However, the study was undertaken in a hospital with extensive experience of this technique for thyroidectomies. Potential complications of a bilateral superficial plexus blockade include local anesthetic toxicity, localized hematoma, and temporary recurrent laryngeal nerve paralysis [9]. 

## 4. Conclusion

Amiodarone is increasingly used for ventricular and supraventricluar arrhythmias. AIT will therefore be encountered more often in patients with cardiac disease. Medical treatment of thyrotoxicosis should be started in first instance in an attempt to render the patient euthyroid. It is desirable for the patient to be euthyroid prior to surgery. However, there is evidence to suggest that general anesthesia can be safely undertaken in patients with thyrotoxicosis and severe cardiac disease.

## Figures and Tables

**Table 1:** Drugs in thyroid treatment and side effects.

Medication	Side effects
Propylthiouracil/Strumazol	Hepatotoxicity, vasculitis, agranulocytosis, anaemia, thrombocytopaenia
Potassium perchlorate	Aplastic anaemia
Corticosteroids	Electrolyte disturbances, cardiac failure, cardiomyopathy, gastric ulcer, diabetes mellitus
Iopanoic acid	Renal impairment, gastrointestinal problems, exacerbation hyperthyroidism
Radioiodine	Hyperthyroidism, hypothyroidism
Beta-blockers	Bronchospasm, hypotension, bradycardia, cardiac failure
